# Supplementary material for: Omega-3 supplements in the prevention and treatment of youth depression and anxiety symptoms: A scoping review
Source: PLoS One. 2023 Apr 20;18(4):e0284057. doi: 10.1371/journal.pone.0284057 (PMC10118139; doi:10.1371/journal.pone.0284057)
Supplement: S2 Table — (DOCX) [file pone.0284057.s002.docx]

**Supplementary Table 1. Search terms for academic databases.**

Search date 4^th^ August 2021

| **Database** | **Search Terms** |
| --- | --- |
| Cochrane  CENTRAL | (“omega-3” OR “n-3” OR “ω-3” OR "polyunsaturated fatty acid*" OR "unsaturated fatty acid*" OR "fatty acid" OR "fatty acids" OR "polyunsaturated fat" OR "polyunsaturated fats" OR "polyunsaturated fatty” OR PUFA* OR "eicosapentaenoic acid" OR "docosahexaenoic acid" OR EPA OR DHA or “fish oil” or “cod liver oil”):ti,ab,kw.  **AND**  (child* or adolesc* or pediatri* or paediatri* or youth or "young person" or "young persons" or "young people" or "young peoples" or "young adult" or "young adulthood" or "young adults" or teen* or student* or high?school or undergrad* or college or university or pubescent):ti,ab,kw  **AND**  (depress* or dysthymi* or "affective disorder*" or "mood disorder*" or "anxiety disorder*" or anxi* or generali?ed anxi* or GAD or "mental health" or phobia* or panic or stress):ti,ab,kw. |
| EmBASE | omega-3 or n-3 or ω-3 or "polyunsaturated fatty acid*" or "unsaturated fatty acid*" or ("fatty acid" OR "fatty acids") or ("polyunsaturated fat" OR "polyunsaturated fats" OR "polyunsaturated fatty") or PUFA* or "eicosapentaenoic acid" or "docosahexaenoic acid" or EPA or DHA or fish oil* or cod liver oil).ti,ab.  **AND**  (child* or adolesc* or pediatri* or paediatri* or youth or ("young person" OR "young persons") or ("young people" OR "young peoples") or ("young adult" OR "young adulthood" OR "young adults") or teen* or student* or high?school or undergrad* or college or university or pubescent).ti,ab.  **AND**  (depress* or dysthymi* or "affective disorder*" or "mood disorder*" or "anxiety disorder*" or anxi* or generali?ed anxi* or GAD or "mental health" or phobia* or panic or stress).ti,ab. |
| PsycINFO | (ti(omega-3 or n-3 or ω-3 or "polyunsaturated fatty acid*" or "unsaturated fatty acid*" or ("fatty acid" OR "fatty acids") or ("polyunsaturated fat" OR "polyunsaturated fats" OR "polyunsaturated fatty") or PUFA* or "eicosapentaenoic acid" or "docosahexaenoic acid" or EPA or DHA or fish oil* or cod liver oil) OR ab(omega-3 or n-3 or ω-3 or "polyunsaturated fatty acid*" or "unsaturated fatty acid*" or ("fatty acid" OR "fatty acids") or ("polyunsaturated fat" OR "polyunsaturated fats" OR "polyunsaturated fatty") or PUFA* or "eicosapentaenoic acid" or "docosahexaenoic acid" or EPA or DHA or fish oil* or cod liver oil))  **AND**  (ti(child* or adolesc* or pediatri* or paediatri* or youth or ("young person" OR "young persons") or ("young people" OR "young peoples") or ("young adult" OR "young adulthood" OR "young adults") or teen* or student* or high?school or undergrad* or college or university or pubescent) OR ab(child* or adolesc* or pediatri* or paediatri* or youth or ("young person" OR "young persons") or ("young people" OR "young peoples") or ("young adult" OR "young adulthood" OR "young adults") or teen* or student* or high?school or undergrad* or college or university or pubescent))  **AND**  (ti(depress* or dysthymi* or ("affective disorder" OR "affective disorders") or ("mood disorder" OR "mood disorders") or ("anxiety disorder" OR "anxiety disorders") or anxi* or “generali?ed anxi*” or GAD or “mental health” or phobia* or panic or stress) OR ab(depress* or dysthymi* or ("affective disorder" OR "affective disorders") or ("mood disorder" OR "mood disorders") or ("anxiety disorder" OR "anxiety disorders") or anxi* or “generali?ed anxi*” or GAD or “mental health” or phobia* or panic or stress)) |
| PubMed | "omega-3"[Title/Abstract] OR "n-3"[Title/Abstract] OR "omega-3"[Title/Abstract] OR "polyunsaturated fatty acid*"[Title/Abstract] OR "unsaturated fatty acid*"[Title/Abstract] OR "fatty acid"[Title/Abstract] OR "fatty acids"[Title/Abstract] OR "polyunsaturated fat"[Title/Abstract] OR "polyunsaturated fats"[Title/Abstract] OR "polyunsaturated fatty"[Title/Abstract] OR "pufa*"[Title/Abstract] OR "eicosapentaenoic acid"[Title/Abstract] OR "docosahexaenoic acid"[Title/Abstract] OR "EPA"[Title/Abstract] OR "DHA"[Title/Abstract] OR "fish oil*"[Title/Abstract] OR "cod liver oil"[Title/Abstract]  **AND**  "child*"[Title/Abstract] OR "adolesc*"[Title/Abstract] OR "pediatri*"[Title/Abstract] OR "paediatri*"[Title/Abstract] OR youth[Title/Abstract] OR "young person"[Title/Abstract] OR "young persons"[Title/Abstract] OR "young people"[Title/Abstract] OR "young peoples"[Title/Abstract] OR "young adult"[Title/Abstract] OR "young adults"[Title/Abstract] OR "young adulthood"[Title/Abstract] OR teen*[Title/Abstract] OR student*[Title/Abstract] OR high?school[Title/Abstract] OR undergrad*[Title/Abstract] OR college[Title/Abstract] OR university[Title/Abstract] OR pubescent[Title/Abstract]  **AND**  "depress*"[Title/Abstract] OR "dysthymi*"[Title/Abstract] OR "affective disorder"[Title/Abstract] OR "affective disorders"[Title/Abstract] OR "mood disorder"[Title/Abstract] OR "mood disorders"[Title/Abstract] OR "anxiety disorder"[Title/Abstract] OR "anxiety disorders"[Title/Abstract] OR "anxi*"[Title/Abstract] OR "generalised anxi*"[Title/Abstract] OR "generalized anxi*"[Title/Abstract] OR GAD[Title/Abstract] OR "mental health"[Title/Abstract] OR "phobia*"[Title/Abstract] OR "panic"[Title/Abstract] OR "stress"[Title/Abstract] |
