# Supplementary material for: Omega-3 supplements in the prevention and treatment of youth depression and anxiety symptoms: A scoping review
Source: PLoS One. 2023 Apr 20;18(4):e0284057. doi: 10.1371/journal.pone.0284057 (PMC10118139; doi:10.1371/journal.pone.0284057)
Supplement: S3 Table — (DOCX) [file pone.0284057.s003.docx]

**Supplementary Table 2. Grey literature search terms and databases**

| **Database** | **Date searched** | **Search terms** |
| --- | --- | --- |
| Substance Abuse & Mental Services Administration | 4.8.21 | omega-3, n-3, ω-3, fatty acid, polyunsaturated fat, fish oil, cod liver oil |
| World Health Organization Regional Office for Europe - Health Evidence Network | 4.8.21 | omega-3, n-3, ω-3, fatty acid, polyunsaturated fat, fish oil, cod liver oil |
| Canadian Agency for Drugs and Technologies in Health | 4.8.21 | omega-3, “ω-3” “polyunsaturated fatty acid” “unsaturated fatty acid" fatty acid” “polyunsaturated fat” "fish oil" |
| International Health Technology Assessment database | 10.8.21 | (omega-3 or n−3 “ω-3” or “polyunsaturated fatty acid*” or “unsaturated fatty acid*” or “fatty acid” or “polyunsaturated fat*” or PUFA* or “eicosapentaenoic acid” or “docosahexaenoic acid” or EPA or DHA or “fish oil*” or “cod liver oil” or “cod-liver oil”) AND (child* or adolesc* or pediatri* or paediatri* or youth or “young people” or “young adult” or teen* or student* or “high school” or “high-school” or undergraduate or college or university or pubescent)  AND (depress* or dysthymi* or “affective disorder*” or “mood disorder*” or “anxiety disorder” or anxiety or anxious or “mental health” or phobia* or panic or stress) |
| National Institute for Health and Care Excellence | 10.8.21 | (omega 3 or "fatty acid" or "fish oil") AND (mental health or anxiety or depression) AND ("young people" or youth or child or adolescent) |
| National Institute for Health Research Innovation Observatory | 10.8.21 | omega-3, n-3, ω-3, fatty acid, polyunsaturated fat, fish oil, cod liver oil |
| Clinical Trials Registry - India | 16.8.21 | omega-3 |
| Open trials (https://explorer.opentrials.net/) | 16.8.21 | (Fish oil OR omega-3 OR fatty acid OR polyunsaturated fat) AND (depression OR mental health OR anxiety) |
| Trip Medical Database | 17.8.21 | Omega-3, fish oil, fatty acid, depression, anxiety, mental health, adolescent, child, youth |
| Google Advanced | 17.8.21 | (omega-3 OR “ω-3” OR "fatty acid" OR "polyunsaturated fat" OR PUFA OR "cod liver oil" OR "fish oil") AND (child OR adolescent OR youth OR "young people" OR "young adult" OR teen OR student) AND (depression OR dysthymia OR anxiety OR mood OR "mental health") |
