# Supplementary material for: Omega-3 supplements in the prevention and treatment of youth depression and anxiety symptoms: A scoping review
Source: PLoS One. 2023 Apr 20;18(4):e0284057. doi: 10.1371/journal.pone.0284057 (PMC10118139; doi:10.1371/journal.pone.0284057)
Supplement: S4 Table — (DOCX) [file pone.0284057.s004.docx]

**Supplementary Table 3. Cochrane Risk of Bias ratings for randomised controlled trials (n=13)**

| Studies | Item 1:  Risk of bias arising from the randomisation process | Item 2:  Risk of bias arising from the effect of assignment to intervention | Item 3:  Risk of bias due to missing outcome data | Item 4:  Risk of bias in measurement of outcome | Item 5:  Risk of bias in selection of the reported result |
| --- | --- | --- | --- | --- | --- |
| Amminger 2010 | Low | Low | Low | Some concerns | Some concerns |
| Amminger 2013 | Low | Low | Low | Low | Some concerns |
| Gabbay 2019 | Some concerns | Low | Some concerns | Low | Low |
| Giles 2015 | Some concerns | Some concerns | Low | Some concerns | Some concerns |
| Ginty 2015 | Low | Low | Low | Low | Some concerns |
| Jamilian 2018 | Low | Some concerns | Low | Low | Some concerns |
| Kiecolt-Glaser 2011 | Low | Low | Low | Low | Low |
| Manos 2018 | Some concerns | Low | Low | Low | Some concerns |
| McGorry 2017 | Low | Low | Low | Low | Low |
| McNamara 2020 | Some concerns | Low | Some concerns | Low | Some concerns |
| Robinson 2019 | Some concerns | Low | Low | Low | Some concerns |
| Trebatická 2020 | Low | Low | Low | Low | Some concerns |
| Van der Wurff 2020 | Some concerns | Low | Low | Low | Low |
