# Supplementary material for: Omega-3 supplements in the prevention and treatment of youth depression and anxiety symptoms: A scoping review
Source: PLoS One. 2023 Apr 20;18(4):e0284057. doi: 10.1371/journal.pone.0284057 (PMC10118139; doi:10.1371/journal.pone.0284057)
Supplement: S5 Table — (DOCX) [file pone.0284057.s005.docx]

**Supplementary Table 4. Ratings of grey literature according to comprehensiveness, accuracy of information, and reference to peer-reviewed literature (n=12)**

| **Sources** | **Comprehensiveness** | **Accuracy of information** | **Reference to peer-reviewed literature** |
| --- | --- | --- | --- |
| Amen Clinics, 2020 | Excellent | Moderate | Excellent |
| British Diet Association, 2020 | Poor | Moderate | Poor |
| Contemporary Paediatrics, 2005 | Poor | Moderate | Moderate |
| Headspace, 2019 | Poor | Moderate | Poor |
| Healthline, 2019 | Moderate | Moderate | Excellent |
| Hey Sigmund, 2017 | Poor | Poor | Poor |
| Kaiser Permanente, 2021 | Excellent | Excellent | Excellent |
| Newport Academy, 2017 | Moderate | Moderate | Moderate |
| Nutri Advanced, 2021 | Excellent | Moderate | Excellent |
| Pediatric Partners, no date | Poor | Moderate | Excellent |
| UNICEF, 2015 | Poor | Poor | Excellent |
| Vital Choice, 2014 | Excellent | Excellent | Excellent |
