# Supplementary material for: Omega-3 supplements in the prevention and treatment of youth depression and anxiety symptoms: A scoping review
Source: PLoS One. 2023 Apr 20;18(4):e0284057. doi: 10.1371/journal.pone.0284057 (PMC10118139; doi:10.1371/journal.pone.0284057)
Supplement: S6 Table — (DOCX) [file pone.0284057.s006.docx]

**Supplementary Table 5. Characteristics of included non-randomised controlled trials (n=4)**

| **Study ID** | **Study type** | **Country** | **Sample size, n** | **Female  participants**  **(%)** | **Demographic** | **Diagnostic criteria** | **Age, mean (SD)** | **Conditions** | **Outcome measure** |
| --- | --- | --- | --- | --- | --- | --- | --- | --- | --- |
| **Amminger, 2015 (38)** | Pilot study | Australia | 68 | 64.7 | Adolescents experiencing moderate to severe depressive symptoms | DSM-IV | 20.1 (2.6) | Omega-3 and placebo (groups were collapsed) | QIDS |
| **Clayton, 2009 (35)** | Uncontrolled open label trial | Australia | 18 | 66.7 | Juvenile bipolar disorder, prescribed with mood-stabilising medication | DSM-IV | F= 16.1 (0.8)  M= 13 (1.1) | Omega-3 (no comparison) | YMRS, HDRS, GASC, CBCL-PR, red blood cell EPA, DPA and DHA levels |
| **Fristad, 2021 (37)** | Observational follow up | United States | 38 | 37.0 | Children with depression participated in OATS study | DSM-IV | 14.6 (2.5) | Those who continued/  initiated omega-3 use following RCT vs. those who did not | CDRS-R, YMRS |
| **McNamara, 2014 (36)** | Open label trial | United States | ~~14~~20 | 60.0 | Adolescents with SSRI-resistant major depressive disorder | DSM-IV | 15.6 (3.2) | High dose vs. low dose omega-3 | CDRS-R, YMRS, erythrocyte fatty acid composition, side effects, blood analyses |

CBCL-PR = Child Behaviour Checklist – Parent Report; CDRS-R = Children’s Depression Rating Scale – Revised; CGIS = Clinical Global Impressions Scale; DHA = docosahexaenoic acid; DPA = Docosapentaenoic acid; DSM-IV = Diagnostic and Statistical Manual of Mental Disorders – Fourth Edition; EPA = eicosapentaenoic acid; F = females; GASC = Global Assessment Scale for Children; HDRS = Hamilton Depression Rating Scale; M = males; OATS = Omega-3 and Therapy study; PRS = parental report of study; QIDS = Quick Inventory of Depressive Symptomatology; SSRI = selective serotonin reuptake inhibitor; YMRS = Young Mania Rating Scale
