# Supplementary material for: Omega-3 supplements in the prevention and treatment of youth depression and anxiety symptoms: A scoping review
Source: PLoS One. 2023 Apr 20;18(4):e0284057. doi: 10.1371/journal.pone.0284057 (PMC10118139; doi:10.1371/journal.pone.0284057)
Supplement: S7 Table — (DOCX) [file pone.0284057.s007.docx]

**Supplementary Table 6. Findings from non-randomised controlled trials investigating the effect of omega-3 supplementation on depression and/or anxiety (n=4)**

| **Study ID** | **Omega-3 daily dose** | **%  EPA, DHA** | **Other interventions**  **(% of participants)** | **Depression** | **Anxiety** | **Other outcomes** | **Side effects and adherence** |
| --- | --- | --- | --- | --- | --- | --- | --- |
| **Amminger, 2015 (38)** | 1180mg for 12 weeks | 59.3% EPA  40.7% DHA | Cognitive-behavioural case management (100) | At end of trial, 25% had clinically relevant depressive symptoms. 75% had  improved clinical symptoms (QIDS). | Not studied | Not studied | Not studied |
| **Clayton, 2009 (35)** | 1920mg  for 6 weeks | 18.8% EPA  81.2% DHA | Lithium (50),  valproate (39),  quetiapine (11)  Risperidone, dexamphetamine, and SSRIs (unknown) | ↓ depression symptoms (HDRS) | Not studied | Significant improvements in global functioning (GASC). Significant ↓ in mania (YMRS),  internalising and externalising symptoms  (CBCL-PR). Increase in red blood cell EPA and DHA levels and decrease in DPA from pre-post treatment. Changes in mood and behaviour were not significantly correlated with red blood cell LCn-3PUFA levels. | 3 participants withdrew because of gastrointestinal disturbance. Mean adherence = 84.8% ± 4.1, estimated by capsule count-back and interviews among study completers |
| **Fristad, 2021 (37)** | Not reported | Not reported | Therapy after RCT (58). Mood stabilisers, anti-obsessionals, antidepressants, anti-psychotics after RCT (63) | ↓ depressive symptoms at follow up for families persisting with omega-3 (CDRS-R) | Not studied | No significant group differences in YMRS scores | Side effects not studied. 50% of follow-up sample reported taking omega-3 after the original RCT. |
| **McNamara, 2014 (36)** | 2400mg (low dose) OR   16200mg (high dose) | 66.7% EPA  33.3% DHA | Fluoxetine (50), citalopram (15), escitalopram (5), sertraline (30) | No significant  change in  depression  symptoms in high dose group  compared to low dose group. ↓ depression  symptoms over time for both groups (CDRS-R). | Not studied | Significant decrease in mania symptoms (YMRS) in high dose group. Change in depression symptoms was uncorrelated with change in EPA and DHA levels. | Headache, difficulty staying asleep, diarrhoea, difficulty waking up, dizziness when standing, appetite increase, dizziness, and joint aches greater in low dose group than high dose group. Nausea, lethargy, abdominal pain, vomiting greater in high dose group compared to low dose group. Mean adherence: low dose (92.0%); high dose (97.0%) |

↑ denotes increase in symptoms; ↓ denotes decrease in symptoms; CDRS-R = Children’s Depression Rating Scale – Revised; DHA = docosahexaenoic acid; DLPFC = dorsolateral prefrontal cortex; EPA = eicosapentaenoic acid; GASC = Global Assessment Scale for Children; Glx = glutamine;  Glu = glutamate; LCn-3PUFA = long-chain n-3 polyunsaturated fatty acid; MDD = Major depressive Disorder; QIDS = Quick Inventory of Depressive Symptomatology; RCT = Randomised Controlled Trial; SSRI = selective serotonin reuptake inhibitor; YMRS = Young Mania Rating Scale
