# Supplementary material for: Omega-3 supplements in the prevention and treatment of youth depression and anxiety symptoms: A scoping review
Source: PLoS One. 2023 Apr 20;18(4):e0284057. doi: 10.1371/journal.pone.0284057 (PMC10118139; doi:10.1371/journal.pone.0284057)
Supplement: Attachment — Submitted filename: Response to reviewers.docx [file pone.0284057.s009.docx]

**Response to reviewers**

**Reviewer #2: My very warm thanks to the authors for their detailed responses to all my comments. I have only one minor amendment suggestion to the text of the paper:

p. 23, line 383-384: Since there were no included studies of moderators, it seems like an over-statement to imply that your findings contrast with those of studies among adults. Absence of studies is different from absence of evidence. Without the evidence, the claim of contrast cannot be upheld. I suggest that the statement be modified as: “However, research conducted among adults suggests that use of omega-3 as an adjunct to antidepressant medication and at higher doses may improve effectiveness thereby serving as moderators of treatment effect” [or something of that nature]. This avoids the burden of contrast while making the valid point.**

We thank the reviewer for their feedback on our manuscript. We have now modified the statement, in line with the author’s suggestion.
